# The Preparation of Patients for Operation

**Published:** 1894-02-24

**Authors:** 


### Medical Progress and Hospital Clinics.

[*The Editor will be glad to receive offers of co-operation and contributions from members of the profession. All letters should be addressed to* The Editor, The Lodge, Porchester Square, London, W.]

### LONDON HOSPITAL.

### The Preparation of Patients for Operation.

The ultimate success of an operation depends to a great extent on the care taken in preparing the patient, often for some considerable time before the operation is actually performed. Of course, a great number of cases have to be operated on with little or no previous preparation, this being the case in injuries requiring immediate operation, and many cases of strangulated hernia; but, though a great number of cases operated on without previous preparation do well, yet in a certain number it is essential, in all advisable.

Many causes militate against the success of an operation, constitutional conditions of the patient being among the most important. The life led just previous and the state of nutrition of the body are all factors of surgical success or failure. Hence the importance of previous knowledge of the patient and treatment of all conditions—physical, hygienic, and mental—that will tend to influence the ultimate success of an operation. With the hygienic surroundings of a patient about to be operated on we have not here to concern ourselves; their importance is now fully understood and attended to. The points that enter into the scope of this article are the application of measures, hygienic or therapeutic, to the patient himself.

Underlying the practice of modern surgery is everywhere the principles of the application of rest, and rest before is as important as rest after operation. In the bustle of modern life the period before an operation is to many a time of extra worry and work to prepare for the term of enforced idleness that must inevitably follow. Body and mind alike overworked are in the worst possible condition to bear the increased strain of an operation, and the result may be failure or tardy and delayed repair.

To obviate this, the period before operation should be one of physical and mental quiet—physical so that the body shall get accustomed to the restraint and irksomeness of bed, the intestinal tract, swept clear of inert and superfluous matter, be brought into regular working order, and the stomach accustomed to regular exercise of its digestive action; in short, that all the animal functions of the body shall be discharged as regularly and easily as possible.

The period before operation should also be one of mental rest, freedom alike from business worry and the obtrusive fears and hopes of sympathetic relatives and friends. True, nervous people often bear operations exceedingly well, but the best state of mind above all others for an operation is the happy-go-lucky one that eats and drinks and takes no thought for the morrow. When the energy of the central nervous system is being expended on doubts and worries as to what is happening, how the case is going to end, or how it is progressing, energy is being used up that in a more placid frame of mind would be acting quietly and regularly on the tissues of the body, carrying on a beneficial system of repair. Often, especially in hospital practice, the previous habits of a patient require serious modification before the most suitable condition for operation is arrived at. The too free use of alcohol is one of great importance; the fêteing by sympathetic friends of the intending subject for operation before his entry into hospital renders necessary a course of blue pill and salines to place his gastro-intestinal tract in a healthy condition. The other main systems of the body require thorough examination; the state of the heart, lungs, kidneys, and vessels must be carefully examined and compared with the real and apparent age of the patient, giving valuable indications as to the dietary, amount and kind of rest, he is likely to require. The presence of chronic bronchitis will be a warning not to keep the patient too long in bed, and not in a prone position, while at the same time it may influence seriously the prognosis of an operation. Degenerate vessels with chronic kidney disease, often associated with secondary cardiac changes, sound a note of warning influencing prognosis, or modifying treatment.

Passing now to the immediate preparation for operation, the patient, whenever possible, having been in hospital for several days previously, the urine tested—daily in doubtful cases—and the heart and lungs examined. The routine practice here is to clear the bowels the night before the operation with a purge of salts and senna, followed by an enema the next morning. The bladder is emptied just before the patient is sent to the operating theatre. The skin is cleaned by a warm bath, then the part immediately involved in the operation cleansed with ether, followed by carbolic water, and a compress of lint soaked in 1 in 30 carbolic, or 1 in 1,000 sublimate solution, applied for twelve hours previous to operation. Any hairs that may be present are removed by shaving before the skin is cleaned. The hair in the case of women plaited, unless the operation is on the head, when the hair must of course be removed. In all cases, especially in children, the warmth of the patient during the operation is an important factor for or against recovery, more so necessarily in prolonged or abdominal operations, where the shock is greater. The patient is clothed, as far as possible, in woollen garments, so arranged that the site of operation and skin around can be easily exposed without uncovering the rest of the body and without impeding the movements of the chest and abdomen in respiration. Long warm stockings, or in children cotton wool bandaged round the lower extremities, are used, and in children a jacket of gamgee tissue is of great use, being warm and yet not restraining the movements of respiration.

The feeding up to the morning of operation is not altered, but no solid food is given for four to six hours before the anæsthetic; beef tea sometimes with a little stimulant is often given with benefit a couple of hours beforehand, but not milk, this being apt to curdle into large masses, which remain in the stomach for many hours, and when vomiting comes on proving a source of embarrassment. Infants often take some considerable training in feeding before it is advisable to subject them to an operation, especially is this the case in infants at the breast suffering from harelip; they have for admission into hospital to be weaned, and taught to take milk from the spoon and bottle before it is safe to operate on them. Often insufficient or unsuitable feeding renders it advisable to defer surgical interference till a better bodily condition has been attained. Few cases require more attention on this point than children with cleft palate and harelip, where milk has to be given with a spoon, the child being kept in an almost upright sitting position while it is being fed, as swallowing is easiest in this position. Very little drug treatment is required in preparation for the operation itself; the few exceptions to this are in eye operations, when the previous instillation of some mydriatic or myotic is sometimes necessary, and occasionally in cases where the operation is on the cerebral cortex, when the previous administration of morphia may be advisable.

